# Supplementary material for: FOXP3+ T cells in uterine sarcomas are associated with favorable prognosis, low extracellular matrix expression and reduced YAP activation
Source: NPJ Precis Oncol. 2021 Nov 19;5:97. doi: 10.1038/s41698-021-00236-6 (PMC8604926; doi:10.1038/s41698-021-00236-6)
Supplement: Supplementary file 1 — Supplementary Information [file 41698_2021_236_MOESM1_ESM.pdf]

Supplementary Information

FOXP3+ T cells in uterine sarcomas are associated with favorable prognosis, low extracellular matrix expression and reduced YAP activation

Okan Gultekin, Jordi Gonzalez-Molina, Elin Hardell, Lidia Moyano-Galceran, Nicholas Mitsios, Jan Mulder, Georgia Kokaraki, Anders Isaksson, Dhifaf Sarhan, Kaisa Lehti, and Joseph W. Carlson

	Leiomyosarcoma	Low grade endometrial stromal sarcoma	Undifferentiated uterine sarcoma	YWHAE-FAM22 translocated sarcoma	Leiomyoma
**Number of cases**	13	16	26	3	13
**Age at diagnosis** (mean, years)	61.4	54.4	62.2	59.1	
**Stage**					
1	6	12	10	3	
2	0	3	3	0	
3	0	1	4	0	
4	4	0	2	0	
N/A	3	0	7	0	
**Time to last follow up** (months)	59.8	96.0	41.6	87.9	
**Status at last follow up**					
Alive	2	13	5	1	
Deceased	11	3	21	2	

Supplementary Table 1

Clinical Data of Cohort including age at diagnosis, disease stage at diagnosis, and survival.

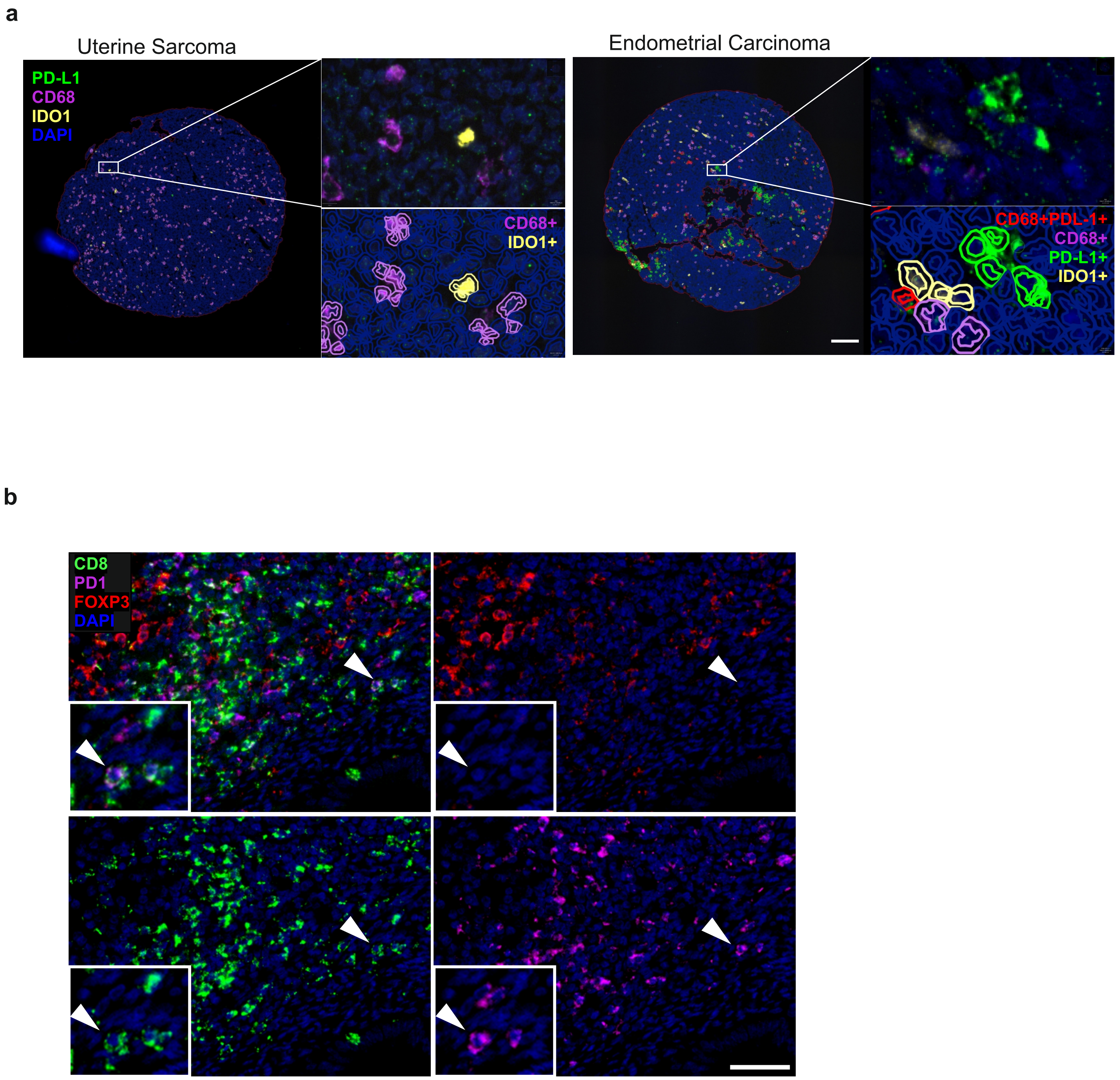

Supplementary Figure 1

PD-L1 expression and CD8+PD1+ cells were detected in control endometrial carcinoma tissues but not in uterine sarcoma. **a**, Example images of PD-L1 staining and QuPath-based cell type detection (bottom right) showing PD-L1+ cells in endometrial carcinoma tissue. Scale bar indicates 100 μm. **b**, Example images of enometrial carcinoma tissue presenting CD8+PD1+ cells. Arrowhead indicates an example of a CD8+PD1+ cell. Scale bar indicates 40 μm.

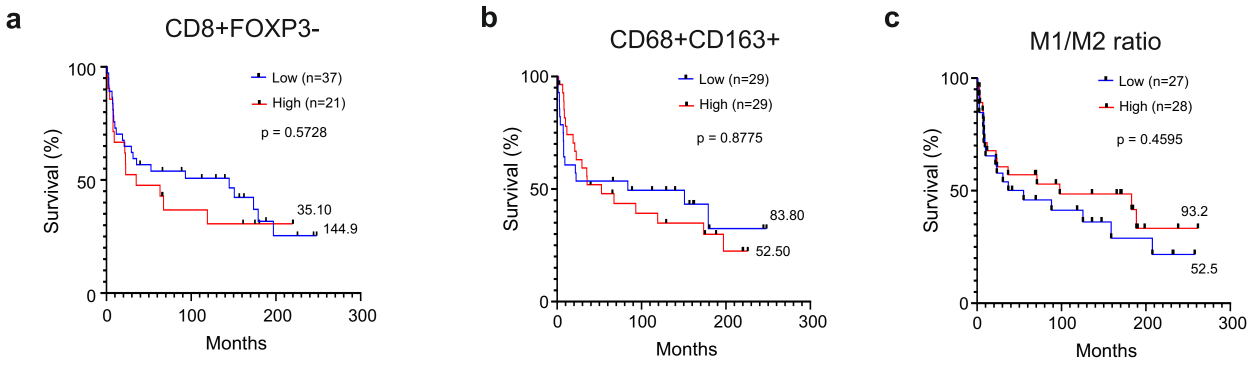

Supplementary Figure 2

Kaplan-Meier curves showing infiltration of CD8+FOXP3- (**a**), M2-like macrophages (CD68+CD163-) (**b**), and M1/M2-like macrophage ratio (**c**), which are not prognostic in uterine sarcomas. Median survival is indicated for each patient group.

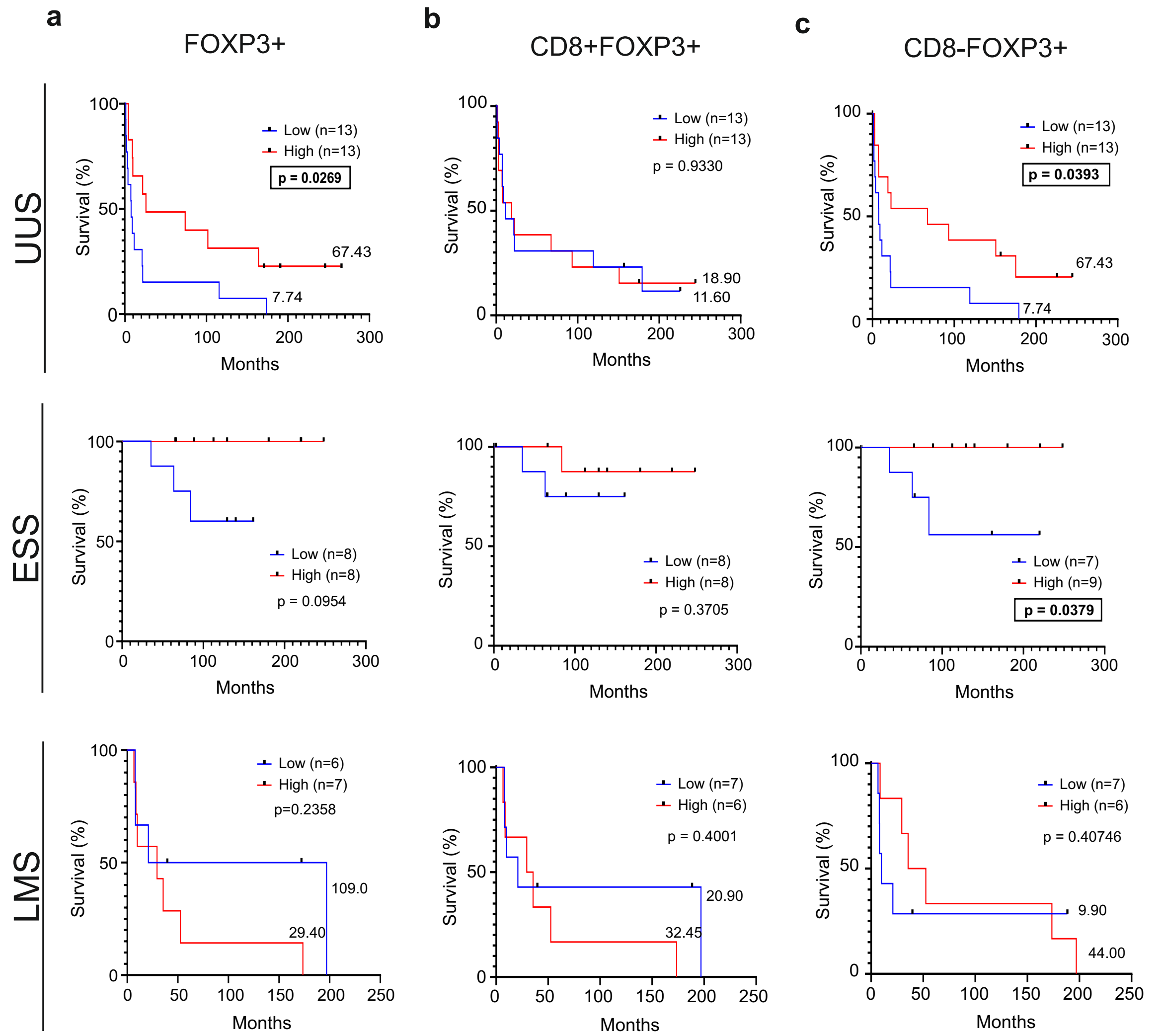

Supplementary Figure 3

FOXP3+ cell density is associated with better survival in UUS and ESS. **a-c**, Kaplan-Meier curves showing overall survival for the indicated FOXP3+ cell subgroups in UUS, ESS, and LMS patients. Median Survival is indicated for each group.

Tumor type	Marker	Group	n	Median survival (months)	P value (log-rank)
UUS	CD68+CD163-	High	13	67.43	**0.0296**
		Low	13	7.737	
	CD68+CD163+	High	14	45.06	0.0509
		Low	12	7.582	
ESS	CD68+CD163-	High	8	Undefined	0.9863
		Low	8	Undefined	
	CD68+CD163+	High	8	Undefined	0.7377
		Low	8	Undefined	
LMS	CD68+CD163-	High	6	22.03	0.7196
		Low	7	29.4	
	CD68+CD163+	High	6	18.9833	0.1283
		Low	7	52.5	

Supplementary Table 2

Summary of median survival and P values for patients grouped according to their M1-like or M2-like macrophage infiltration in ESS, UUS, and LMS tumors.

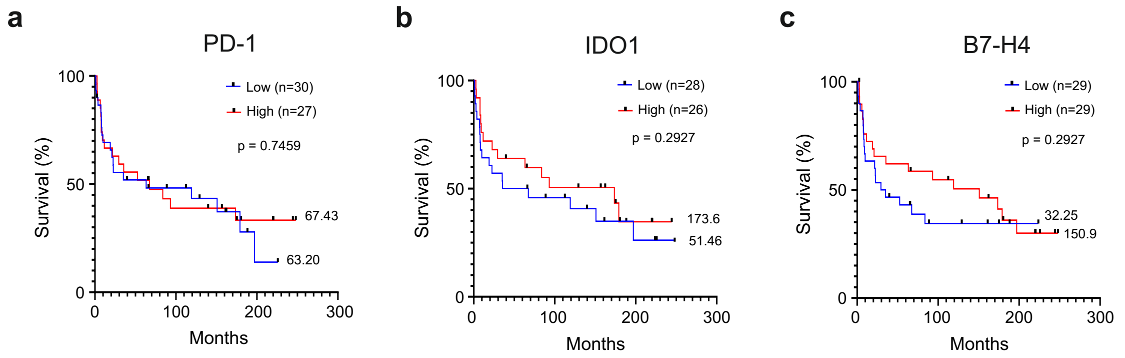

Supplementary Figure 4

Kaplan-Meier curves of patient groups based on expression of immune regulatory proteins PD-1 (**a**), IDO1 (**b**), and B7-H4 (**c**) showing that the general expression of these proteins does not have prognostic value in uterine sarcomas. Median Survival is indicated for each group.

Tumor type	Marker	Group	n	Median survival (months)	P value (log-rank)
UUS	IDO1	High	12	17.15	0.2063
		Low	13	7.427	
	B7H4	High	13	93.21	0.0606
		Low	13	7.737	
	PD-1	High	13	21.59	0.7571
		Low	13	9.197	
ESS	IDO1	High	9	Undefined	0.47
		Low	7	Undefined	
	B7H4	High	8	Undefined	0.6148
		Low	8	Undefined	
	PD-1	High	7	Undefined	0.4319
		Low	9	Undefined	
LMS	IDO1	High	5	173.567	0.3577
		Low	5	9.9	
	B7H4	High	7	20.9	0.6169
		Low	7	29.4	
	PD-1	High	6	44	0.5934
		Low	7	9.9	

Supplementary Table 3

Summary of median survival and P values for patient grouped according to their immune regulatory protein expression in ESS, UUS, and LMS tumors.

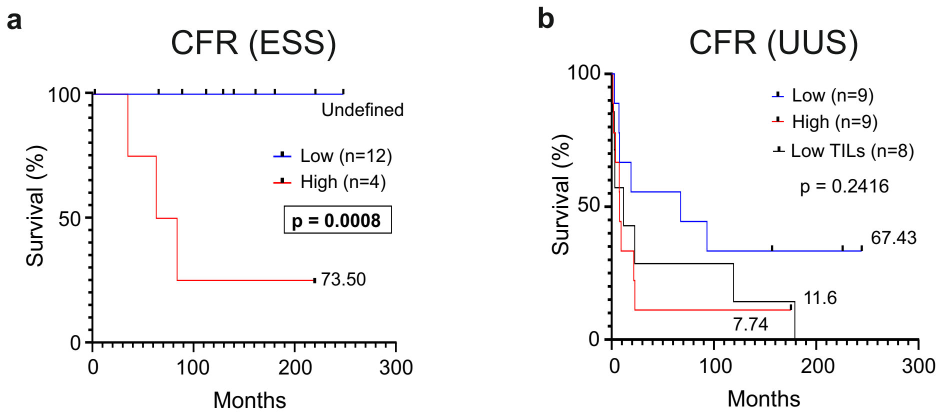

Supplementary Figure 5

High CD8+FOXP3-/FOXP3+ ratio (CFR) is associated with poor survival. Kaplan-Meier curves of ESS (**a**) and UUS (**b**) patients grouped based on their CFR. UUS tumours include a low-TIL group defined as tumors with T cell infiltration below the 40th percentile of all tumors analyzed. Median Survival is indicated for each group.

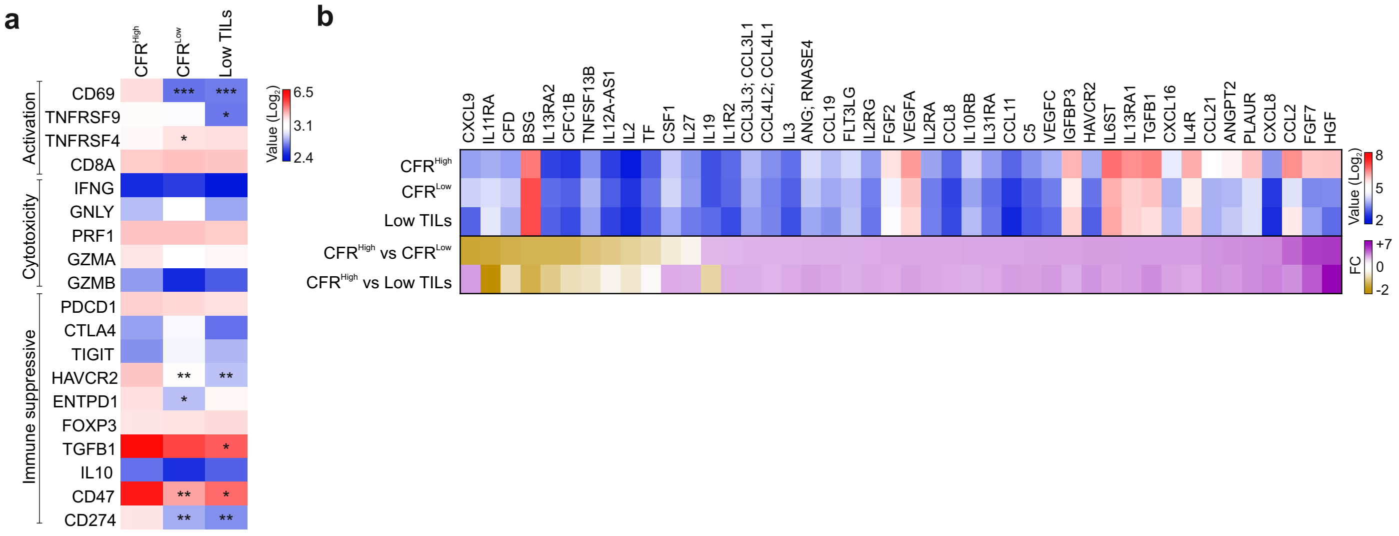

Supplementary Figure 6

Differential expression of immune marker (**a**) and cytokine genes (**b**) between CFR^{High} and CFR^{Low} or Low TILs (FC, fold change). Significance is indicated as *(P< 0.05), ** (P<0.01), *** (P<0.001)

a

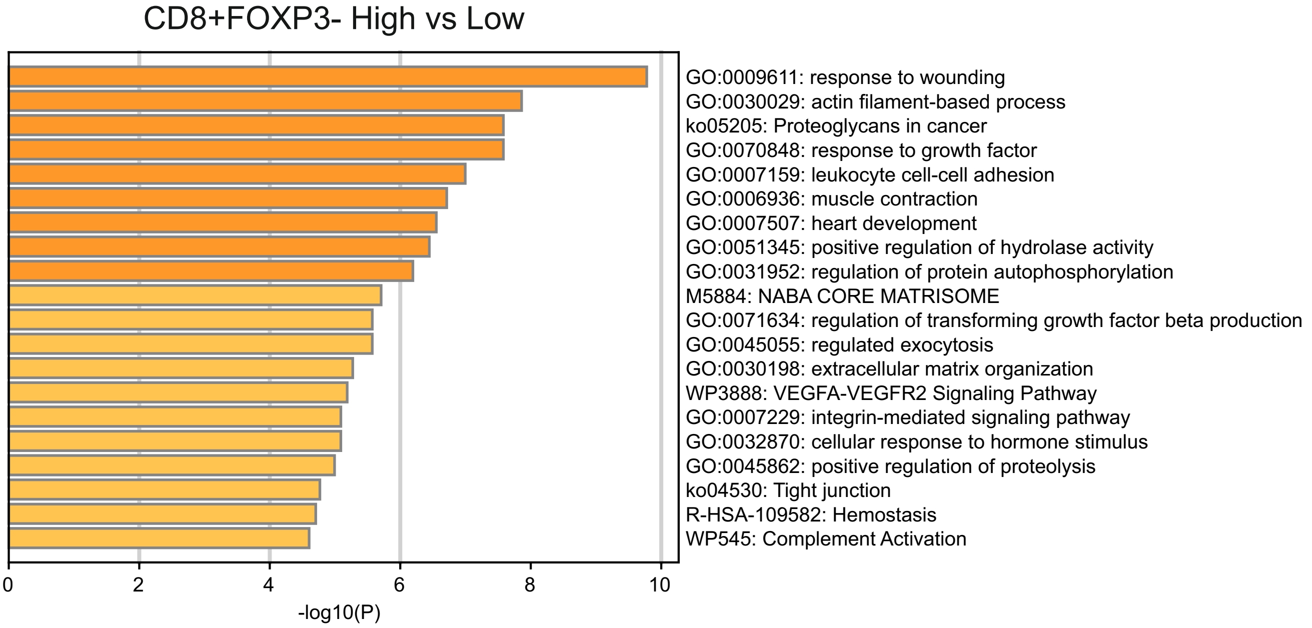

b

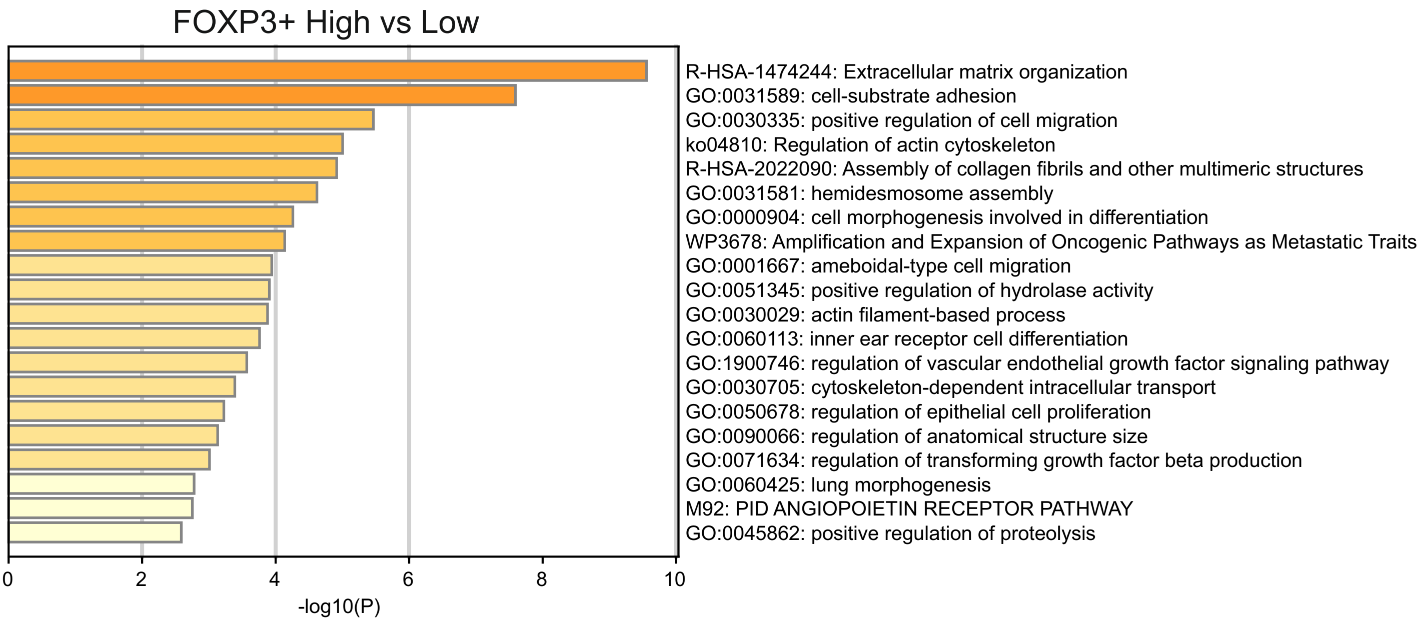

Supplementary Figure 7

Tumors with distinct CD8+ and FOXP3+ cell infiltration show differential expression of extracellular matrix-related genes. **a**, **b,** Metascape pathway analysis reveals the most significantly altered pathways between tumors with high and low CD8+ cell infiltration (**a**), and FOXP3+ cell infiltration (**b**).

Antibody	Company	Product No.	Dilution	Technique
CD8	Dako	M 7103	1 : 100	IF
FOXP3	EuroMAbNET	236A/E7	none	IF
PDCD1 / CD279 (PD1)	HPA	HPA035981	1 : 250	IF
CD68	Dako	M 0876	1 : 100	IF
CD163	HPA	HPA046404	1 : 1600	IF
PDL1	Dako	M 3653	1 : 100	IF
IDO1	HPA	HPA023149	1 : 50	IF
VTCN1 (B7-H4)	HPA	HPA054200	1 : 70	IF
CD4	Ventana	790-4423	none	IHC
Collagen I	Abcam	ab34719	1 : 200	IHC
Collagen VI	Abcam	ab6588	1 : 200	IHC
Fibronection	Sigma	F3649	1 : 200	IHC
MMP14	Millipore	MAB3328	1 : 100	IHC
YAP1	Abcam	ab56701	1 : 1000	IHC

Supplementary Table 4

Summary of the details of the antibodies used for immunofluorescence (IF) and immunohistochemistry (IHC). Human protein atlas is represented as HPA.

Supplementary Data 1

List of differentially expressed genes (> 2-fold) between UUS tumors based on CFR groups and Low TILS

Supplementary Data 2

List of differentially expressed genes between UUS tumors with high and low infiltration of M1-like macrophages.
