# Supplementary material for: FOXP3+ T cells in uterine sarcomas are associated with favorable prognosis, low extracellular matrix expression and reduced YAP activation
Source: NPJ Precis Oncol. 2021 Nov 19;5:97. doi: 10.1038/s41698-021-00236-6 (PMC8604926; doi:10.1038/s41698-021-00236-6)
Supplement: Supplementary file 4 — Reporting Summary [file 41698_2021_236_MOESM4_ESM.pdf]

nature portfolio

Corresponding author(s): Joseph W Carlson

Last updated by author(s): Jun 24, 2021

Reporting Summary

Nature Portfolio wishes to improve the reproducibility of the work that we publish. This form provides structure for consistency and transparency in reporting. For further information on Nature Portfolio policies, see our Editorial Policies and the Editorial Policy Checklist.

Statistics

For all statistical analyses, confirm that the following items are present in the figure legend, table legend, main text, or Methods section.

n/a	Confirmed	
☐	☒	The exact sample size (*n*) for each experimental group/condition, given as a discrete number and unit of measurement
☐	☒	A statement on whether measurements were taken from distinct samples or whether the same sample was measured repeatedly
☐	☒	The statistical test(s) used AND whether they are one- or two-sided<br>*Only common tests should be described solely by name; describe more complex techniques in the Methods section.*
☐	☒	A description of all covariates tested
☐	☒	A description of any assumptions or corrections, such as tests of normality and adjustment for multiple comparisons
☐	☒	A full description of the statistical parameters including central tendency (e.g. means) or other basic estimates (e.g. regression coefficient) AND variation (e.g. standard deviation) or associated estimates of uncertainty (e.g. confidence intervals)
☐	☒	For null hypothesis testing, the test statistic (e.g. *F*, *t*, *r*) with confidence intervals, effect sizes, degrees of freedom and *P* value noted<br>*Give P values as exact values whenever suitable.*
☒	☐	For Bayesian analysis, information on the choice of priors and Markov chain Monte Carlo settings
☒	☐	For hierarchical and complex designs, identification of the appropriate level for tests and full reporting of outcomes
☐	☒	Estimates of effect sizes (e.g. Cohen's *d*, Pearson's *r*), indicating how they were calculated

Our web collection on statistics for biologists contains articles on many of the points above.

Software and code

Policy information about availability of computer code

Data collection	QuPath v.0.2.0-m2 was used
Data analysis	R v.4.0.3 (R Core Team, 2020) with heatmap.2 package.<br>GraphPad Prism v8.0

For manuscripts utilizing custom algorithms or software that are central to the research but not yet described in published literature, software must be made available to editors and reviewers. We strongly encourage code deposition in a community repository (e.g. GitHub). See the Nature Portfolio guidelines for submitting code & software for further information.

Data

Policy information about availability of data

All manuscripts must include a data availability statement. This statement should provide the following information, where applicable:

- Accession codes, unique identifiers, or web links for publicly available datasets
- A description of any restrictions on data availability
- For clinical datasets or third party data, please ensure that the statement adheres to our policy

Affymetrix expression array, and clinical metadata are available via the Gene Expression Omnibus database repository accession number GSE119043

Field-specific reporting

Please select the one below that is the best fit for your research. If you are not sure, read the appropriate sections before making your selection.

☒ Life sciences ☐ Behavioural & social sciences ☐ Ecological, evolutionary & environmental sciences

For a reference copy of the document with all sections, see nature.com/documents/nr-reporting-summary-flat.pdf

Life sciences study design

All studies must disclose on these points even when the disclosure is negative.

Sample size	Sample size was limited by the rarity of these tumors. In total, 58 malignant uterine sarcoma cases were included together with 14 benign tumors (leiomyomas)
Data exclusions	N/A
Replication	Duplicates of tissue microarray (TMA) cores per patient were used
Randomization	N/A
Blinding	N/A

Reporting for specific materials, systems and methods

We require information from authors about some types of materials, experimental systems and methods used in many studies. Here, indicate whether each material, system or method listed is relevant to your study. If you are not sure if a list item applies to your research, read the appropriate section before selecting a response.

Materials & experimental systems

n/a	Involved in the study	
☐	☒	Antibodies
☒	☐	Eukaryotic cell lines
☒	☐	Palaeontology and archaeology
☒	☐	Animals and other organisms
☐	☒	Human research participants
☒	☐	Clinical data
☒	☐	Dual use research of concern

Methods

n/a	Involved in the study	
☒	☐	ChIP-seq
☒	☐	Flow cytometry
☒	☐	MRI-based neuroimaging

Antibodies

Antibodies used	CD8 (Dako, M 7103); FOXP3 (EuroMAbNET, 236A/E7); PDCD1/CD279 (PD1) (HPA, HPA035981); CD68 (Dako, M 0876); CD163 (HPA 8, HPA046404); PDL1 (Dako, M 3653); IDO1 (HPA, HPA023149); VTCN1 (B7-H4) (HPA, HPA054200); CD4 (Ventana, 790-4423); Collagen I (Abcam, ab34719); Collagen VI (Abcam, ab6588); Fibronection (Sigma, F3649); MMP14 (Millipore, MAB3328); YAP1 (Abcam, ab56701)
Validation	All antibodies used in this study are commercially available and have been validated for the application by the manufacturer or by specific references found on the suppliers homepage.

Human research participants

Policy information about studies involving human research participants

Population characteristics	This study includes a total of 72 female patients including 13 leiomyosarcomas, 16 endometrial stromal sarcomas, 26 undifferentiated uterine sarcomas, 3 YWHAE-FAM22 endometrial stromal sarcomas, and 14 benign leiomyomata controls. Patients had not received any prior treatment. Complete clinical information can be found in Supplementary Table 1.
Recruitment	Samples were obtained from the Stockholm medical biobank (Swedish biobank reg nr: 914). Specimen collection within the biobank: ”Biobank for molecular biological, functional and clinical studies of gynecologic tumors BBK 1615. All samples were obtained from necessary operations. For eliminating biases, all samples were handled with coded ID-numbers given immediately after sample collection.
Ethics oversight	Ethical application submitted to the Swedish ethical board describing the consent form to be used and the ethical issues involved.

Approval from the ethical board of the submitted ethical application (approval Dnr 03-384 (2003-12-02) 2016/1197-31/1).
Addendum to the ethical board specifying the particular details of the study examining sarcoma tissue, and the proposed analyses (approval stamped on same letter 2018/118-32).
Biobank approval to collect these samples (BBK1615)

Note that full information on the approval of the study protocol must also be provided in the manuscript.
